# The impact of self-avatars on trust and collaboration in shared virtual environments

**DOI:** 10.1371/journal.pone.0189078

**Published:** 2017-12-14

**Authors:** Ye Pan, Anthony Steed

**Affiliations:** Virtual Environments & Computer Graphics Group, Department of Computer Science, University College London, London, United Kingdom; University of Muenster, GERMANY

## Abstract

A self-avatar is known to have a potentially significant impact on the user’s experience of the immersive content but it can also affect how users interact with each other in a shared virtual environment (SVE). We implemented an SVE for a consumer virtual reality system where each user’s body could be represented by a jointed self-avatar that was dynamically controlled by head and hand controllers. We investigated the impact of a self-avatar on collaborative outcomes such as completion time and trust formation during competitive and cooperative tasks. We used two different embodiment levels: no self-avatar and self-avatar, and compared these to an in-person face to face version of the tasks. We found that participants could finish the task more quickly when they cooperated than when they competed, for both the self-avatar condition and the face to face condition, but not for the no self-avatar condition. In terms of trust formation, both the self-avatar condition and the face to face condition led to higher scores than the no self-avatar condition; however, collaboration style had no significant effect on trust built between partners. The results are further evidence of the importance of a self-avatar representation in immersive virtual reality.

## Introduction

Consumer virtual reality systems based on head-mounted displays (HMDs) are now widely available. These systems can include hand-held input devices to enable navigation and interaction in the virtual world. Most applications only display a partial representation of the user, such as the controllers or models of the hands (e.g. toybox demo for oculus touch [2]), in the virtual environment (VE). Relatively few applications include a full body self-avatar. However, as reviewed in more detail in the next section, a self-avatar has been shown to have a positive benefit to interaction tasks, the sense of presence and perceptual judgments [10].

We developed a virtual reality system where several users can meet and interact in a shared virtual environment. Each user has a self-avatar. Each user wears a HTC Vive HMD and can see themselves from a first person view. Each user’s head and hands are tracked via the HTC Vive’s tracking system. The body movements of each user are transmitted through a server such that everyone can see each other’ avatars and feel their presence in the virtual world.

We investigated whether the presence of a self-avatar can affect collaborative outcomes in SVE. Many previous studies showed that a self-avatar can aid in general performance of tasks, as it becomes easier to share information (e.g. [35, 44]). However, there are also situations, particularly in competitive gaming, where participants may not wish to share all their information with each other but still need to check their partner’s progress for strategic purposes. Thus, it is important to understand the impact of a self-avatar not only for cooperative tasks, but also for competitive tasks. We designed an experiment to compare two virtual embodiment levels with a face to face condition. In the first virtual embodiment condition, a no self-avatar condition, where each user was represented only by models of controllers. This representation is common in consumer applications at the moment. In the second embodiment condition, self-avatar condition, each user represented by a full body avatar. The avatar was animated using the tracking data and inverse kinematics. Finally, the face to face condition provided a benchmark to understand avatar mediated interaction. Participants performed two tasks. They performed the task in both competitive and cooperative collaboration modes. We used quantitative and qualitative data analysis to examine users’ performance.

We found that participants completed the task more quickly in a cooperative collaboration style than they did using a competitive collaboration style in the face to face condition and self-avatar condition; but no significant effect was found in the no self-avatar condition. The experimental results also demonstrated that, after completing competitive and cooperative tasks, participants in the face-to-face condition and the self-avatar condition would show higher levels of trust than the no avatar condition. However, no significant effect was found due to the collaboration style. Furthermore, observational results revealed a self-avatar could help to occlude their personal information during competitive task, convey obvious cues about the user’s location and non-verbal information to aid collaborations, and increase the amount of trust built between partners. Together, the results suggest that by simply adding a self-avatar inside an current consumer head-mounted display systems could influence collaborative outcomes. This has implications for future system designs.

In the following sections, we review related work in the area of SVE, self-avatars, embodiment and computer-supported cooperative work. Section 3 contains a description of the system implementation. The experiment is covered in Section 4. Finally, we present discussions of the results, conclusions and future work.

## Related work

### Collaborative VR

Shared virtual environments (SVEs) have been a topic of research for many years in virtual reality (e.g., [9, 40, 41]). An SVE is a virtual environment that supports multiple users that are geographically distributed. A detailed overview of the area is beyond the scope of this paper and we describe a subset of the work that concerns consumer virtual reality systems and impact of the self-avatar. A good overview can be found in [8].

With the launch of Oculus Rift, HTC Vive and Samsung Gear VR, several lightweight, easily configurable SVE systems and applications have been developed. For example, MuVR [6] used a Oculus Rift HMD and a Razer Hydra tracking system, and created multiuser virtual reality platform. MetaSpace [7] included a self-avatar by tracking each user’s body with a Kinect device.

Inspired by these recently developed systems, we developed an SVE system and provided a self-avatar for each user, without depending on any additional tracking system. We further investigated the impact of the use of a self-avatar inside an SVE.

### Self-avatar or no self-avatar

Relatively few HMD-based virtual environment systems display a rendering of the user’s own body. Subjectively, this often leads to a sense of disembodiment in the virtual world. A recent paper by Biocca reviews the potential pros and cons of using a self-avatar [17].

The impact of a self-avatar has been extensively investigated, including the visual embodiment of the user, means of interaction with the world, means of sensing various attributes of the world etc [5, 14]. As described in detail previously [15], some authors have looked at the mechanisms by which the self-avatar embodies the user, thus the user can experience the self-avatar as their own body [15, 22]. Yuan et al. showed that the association between virtual limb and body could be made by the participant engaging in an interactive task in a HMD VR system [13]. Several studies have looked at the influence of a self-avatar on spatial awareness [19, 21]. Mohler et al. showed that an animated self-avatar was superior to a static self-avatar in distance estimation tasks [21].

The self-avatar in an SVE has crucial functions in addition to those it has in a single-user virtual environment [12, 16, 23]. These functions included perception (to see if anyone is around), localisation (to see where the other person is), identification (to recognise the person), visualisation of others’ interest focus (to see where the person’s attention is directed), visualisation of others’ actions (to see what the other person is doing and what is meant through gestures) and social representation of self through decoration of the avatar (to know what the other participants’ task or status is). For example, McManus et al. found that participants performed the tasks faster and more accurately when they either had a self-avatar or saw a character animation [18]. Dodds et al. have shown that a self-avatar is a useful resource in communicating with another person within a multi-user virtual reality system [20].

Additionally, several papers from Bailenson’s group explore how avatar representations can affect users’ behaviour [45, 46]. For example, they found that people used less force touching female avatars than male avatars [45]. Participants assigned to more attractive avatars were more intimate with confederates in a self-disclosure and interpersonal distance task than participants assigned to less attractive avatars. Participants assigned to taller avatars behaved more confidently in a negotiation task than participants assigned to shorter avatars [46].

The general thrust of the work indicates that self-avatars are important and that animation of the avatar can improve the effect of the self-avatar for most cooperative tasks within the virtual environment. It is not so clear what the role of a self-avatar might be for competitive tasks that might require certain information to remain private. We aim to grow the existing knowledge on how the self-avatar alters the collaboration outcomes in an SVE.

### Cooperation, competition and trust

The type of task completed in the SVE may also affect the performance elicited by different level of embodiments. Competitive or cooperative tasks are likely to show a greater difference on variables such as physiological response, aggression, or perceptions of the avatar.

Several studies have demonstrated that task type could be an important factor [42, 43]. Bolton et al. investigated how different interactive large display form factors can support differences in sharing of information during competitive and cooperative task conditions [11]. They suggested that a large flat display had significant advantages for cooperative tasks compared to a spherical display. This was in part because the spherical display supported less mutual awareness of because part of the spherical display might be hidden from one or more viewers. Fox et al. studied whether agents and avatars in virtual environments elicit different levels of social influence [24].

They found that the effect of agency is amplified within competitive and cooperative tasks rather than neutral tasks. In this study, we therefore developed two different scenarios: one cooperative and one competitive.

To further evaluate participants’ collaboration outcomes, we also looked at development of trust. As a measure of trust, as described in detail previously [47], social dilemma games, such as Daytrader, have been used in a variety of experimental studies [25–27].

Additionally, among HCI scholars, there is particular concern about the differences in objective and subjective measures in virtual environments [29]. Objective measures include behavioral data collected by the experimenter or other devices. Subjective measures are those that are self-reported and rely on the participant to provide accurate assessments of their experiences. Thus, these measures may be less reliable because they are subject to participants’ biases. For example, Bailenson et al. demonstrated that behavioral measures could be sensitive enough to pick up responses to digital representations, such as personal distance, that self-reports could not [28]. Given these mixed findings, we have included both subjective and objective measures.

## Technical setup

**Fig 1:**
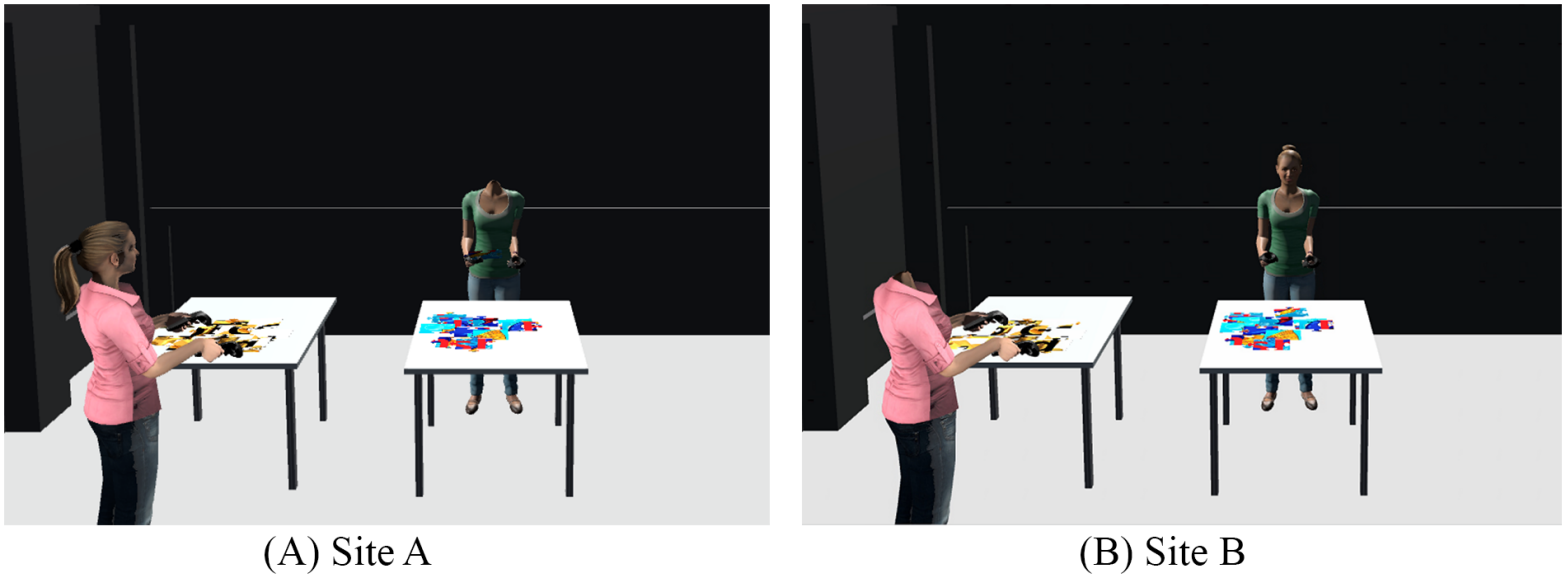
Experimental setup at two sites taken from the same viewpoint. Virtual model showing background, tables, headless local avatar, remote avatar and jigsaw puzzle pieces.

In this section we discuss the system design and implementation of the experiment application. The experiment were conducted at two very similar sites on the same floor of a building. These two sites were networked, so that the users were physically separated while working together in the virtual environment (see Fig 1). In both sites, users were represented by avatars. The movement of the users was free within the limit of the room.

Each user was supported by a different VE system. Each VE system on a Windows 8.1 computer with an Intel Core i7 processor, 8GB ram and a GeForce TitanX graphics card. We provided each user with a HTC Vive to view the virtual world and control the self-avatar. The virtual environment was created using Unity and written in C#. All scenes were rendered at 90Hz.

### Scene, avatars, and puzzles

The scene consisted of three elements: a background scene, self-avatars, and the Jigsaw Phrase Guessing game materials.

The background scene was a model of the physical site where the experiment took place for the face to face condition. The physical site is a 446cm × 618 cm room with black walls (see Fig 1). The virtual model copies these features. For this experiment we placed two tables in the room, and modeled the tables in the virtual scene in the same size and position.

Some participants had a self-avatar. We provided both male and female avatars in generic clothing, taken from the Rocketbox Complete Characters HD set. The self-avatars were animated using the Final IK plugin from RootMotion [1]. We used the Full Body IK (FBIK) system for biped characters from this plugin. Instead of using single IK handles for each limb, FBIK let us translate and rotate a avatar’s body parts with FBIK effectors and make the rest of the body follow those movements. For example, if we used FBIK to animate the arm of a biped avatar reaching down to pick up an object off the floor, the avatar would bend naturally at the waist and knees as we move the avatar’s hand effectors downward. For hand tracking, we used HTC Vive controllers. We then linked the avatar’s hand effectors to the controller, to create natural poses and animation. Also, since FBIK does not have effectors for the fingers and toes, we manually posed the fingers holding the controller. The avatar’s hand shape was static throughout the experience. We used the HTC Vive headset’s own head-tracker to infer the approximation of the invisible head and torso movements. Each participant’s height was used to scale the size of avatars.

For rendering purposes, the self-avatars were modelled without a head for the local scene (see green shirt avatar in Fig 1A). Thus when the participant looked straight down they would see the torso and limbs of the self-avatar.

The final elements of the scene, the Jigsaw Phrase Guessing game materials, are also described in the next section (see Section Jigsaw Phrase Guessing Game). We provided two images for each round. In the real environment, each image was printed onto 40cm × 60cm cardboard, cut into 12 jigsaw puzzle pieces and scattered on each table. By using Puzzle Maker [4], we created the jigsaw puzzle pieces in the virtual scene in a similar manner. The images would also appear 40cm × 60cm size on the table. This was necessary to ensure that the pieces could be easily visible and manipulable in the HMD.

### Manipulating objects

We implemented an object interaction system by using SteamVR Unity Toolkit [3]. In the VE, by touching a jigsaw puzzle piece using the Vive controller and pressing the trigger, users could grab and snap the piece to the controller. The piece would be released when the trigger was released. When the trigger was released, the piece was propelled in the direction and at the velocity the controller was moving.

### Networking

**Fig 2:**
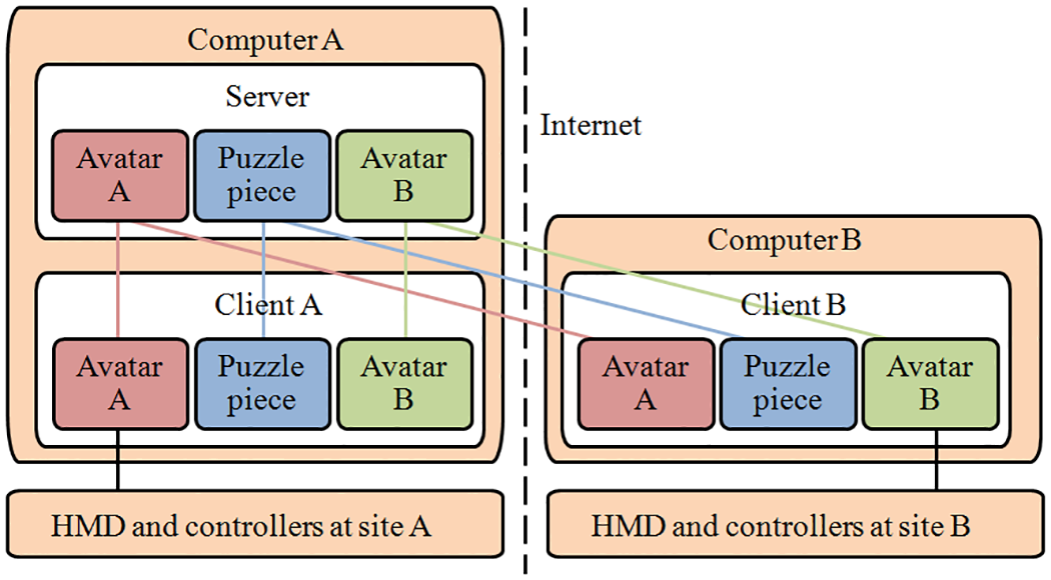
The networking diagram of our multiuser environment.

For a synchronous multiuser virtual experience, it is essential that all persons receive the same state for the virtual environment. We implemented a client-server system using the Unity UNET system (see Fig 2). For example, to sync the movement of the user A, we first tracked his or her physical movement using HTC Vive, and obtained three 3D coordinate frames (left controller, right controller, and head) to animate the avatar A at the client site A. Then, these three 3D coordinates were submitted to the server, and propagated to all the clients. At the client site B, the avatar A would be animated base on these three 3D coordinate frames.

We focus solely on the visual components of the system, as aural communication is supported using Skype. We identify spatialized 3D audio as an area of future work.

## Experiment

We conducted a controlled laboratory experiment to compare the impact of self-avatars on cooperative and competitive collaboration. The experiment was approved by University College London Research Ethics Committee, project ID number 4547/004.

### Hypotheses

**Fig 3:**
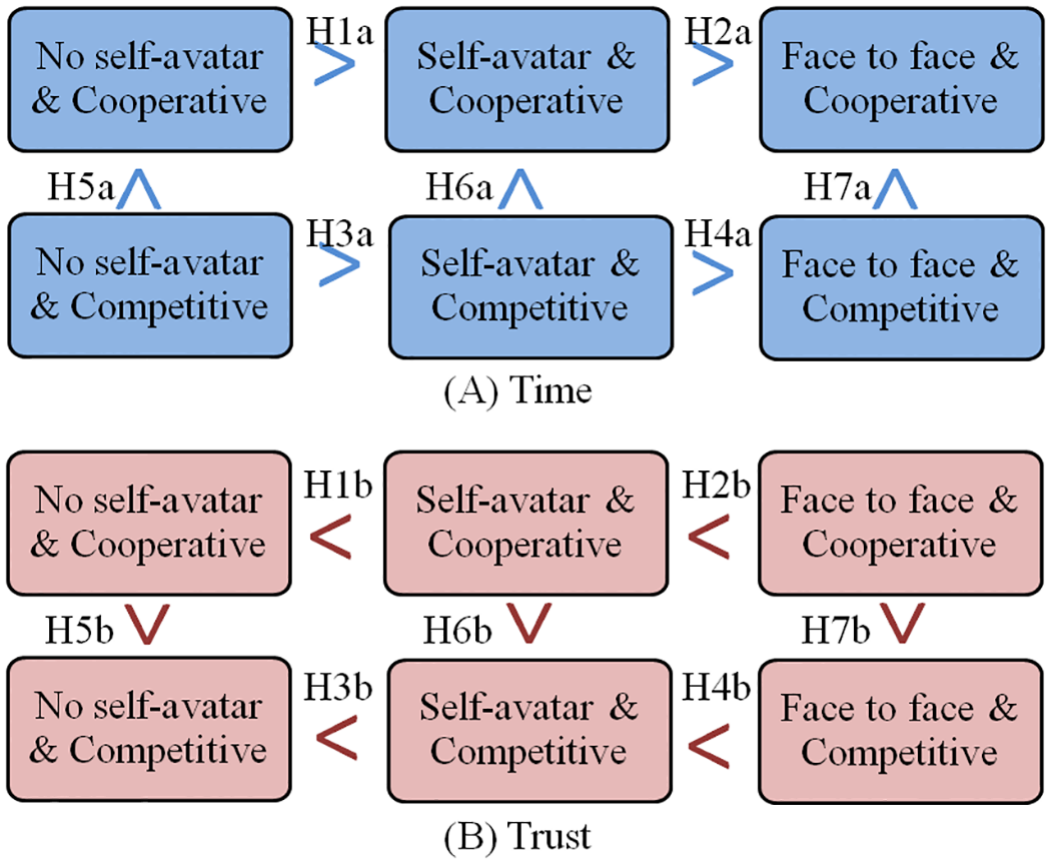
Overview of hypotheses. Note: H1a is for hypothesis 1a, completing the cooperative tasks without a self-avatar will require more time than with a self-avatar. H1b is for hypothesis 1b, after completing the cooperative tasks, the no self- avatar condition will demonstrate lower levels of trust than the self-avatar condition.

Fig 3 presents an overview of hypotheses. We expect that face to face condition will result in more positive collaborative outcomes, followed by the self-avatar condition and then the no self-avatar condition (H1a, H2a, H3a, H4a, H1b, H2b, H3b, H4b in Fig 3). We further expect participants will result in more positive collaborative outcomes in a cooperative style than they did using a competitive style (H5a, H6a, H7a, H5b, H6b, H7b in Fig 3).

### Method

#### Participants

48 participants, 24 dyads, were recruited to take part as users via University College London Subject Pool, bulletin boards at University College London, e-mail lists, and in-person enlistment. Ages ranged from 18-42 (*M* = 27.95, *SD* = 6.43), 50% were male. All pairs were unacquainted with each other before the study, and never interacted with each other except via their particular experimental condition. Participants were not allowed to exchange social information either before or during the game.

#### Material

Participants performed two main tasks: the Daytrader game and the Jigsaw Phrase Guessing game. These tasks were chosen because they provided multiple measures of task performance.

**Daytrader game** Our use of the Daytrader Game was inspired by previous work [25–27]. The game involved three sets of five rounds. For each set of the Daytrader Game, each participant was given 30 credits that they could either keep or put into a pool that was shared between the two participants. At the end of the round, credits that they chose to keep doubled in value, while the credits in the shared pool tripled and were then split evenly between the two participants. At the end of each set of five rounds, the participant earning the most credits in that set received a 300 credits bonus. This bonus had the effect of giving a extra profit to the participant who contributed less than his or her partner. If both participants earned the same amount, they both received the bonus.

**Fig 4:**
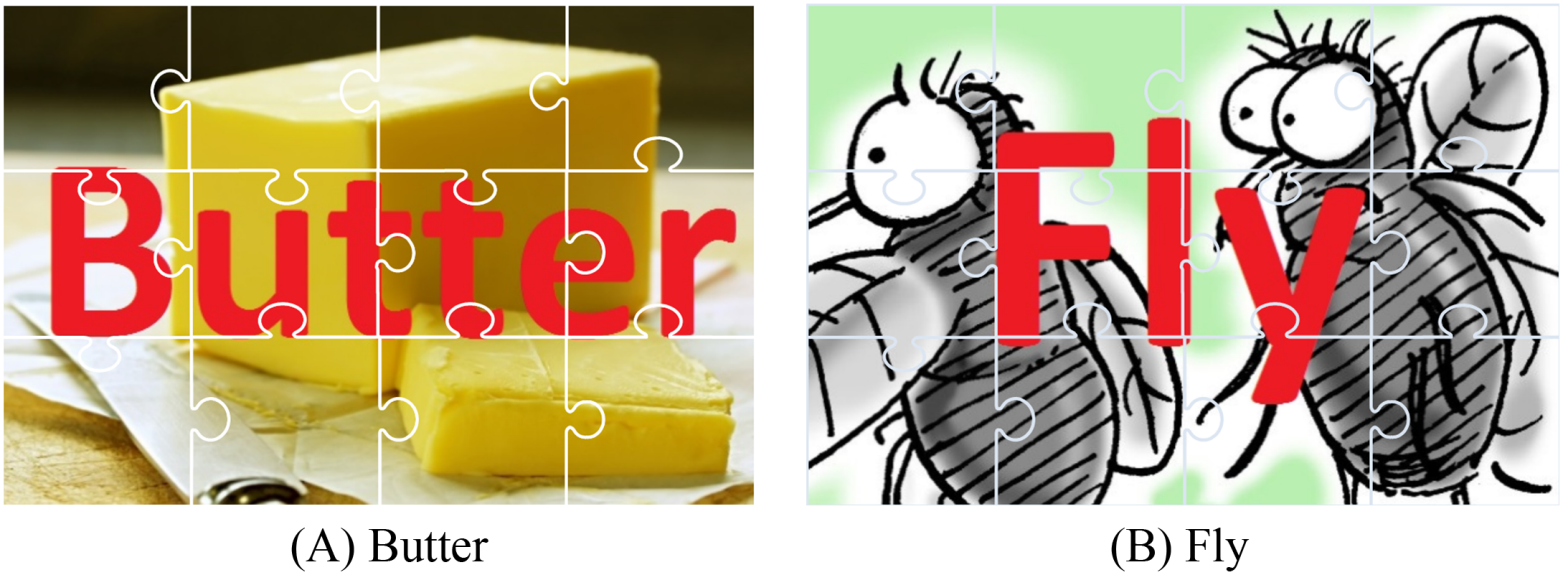
A pair of images for the Jigsaw Phrase Guessing game. (word: butter + word: fly = phrase: butterfly).

**Jigsaw Phrase Guessing game** Our use of the Jigsaw Phrase Guessing game followed the structure employed by previous work [11]. We modified the basic jigsaw puzzle game by combining it with a phrase guessing game in order to increase the utility of peeking and communication between participants. The goal of our modified jigsaw puzzle game was to find a hidden two-word phrase inside two solution images (example see Fig 4). Each solution image contained a different word, which together formed a phrase. There were two components to completing a game: finding a word and finding a phrase. When a participant moved a piece to the correct position, this piece might reveal a portion of a word. Participants would not need to assemble all pieces of the jigsaw puzzle if they collaborated and guessed the phrase based on a partially solved puzzle. Both participants were placed at the centre of the room, started with the same puzzle displayed on two tables in the real or virtual environment. They were able to use both hands to move pieces, walk around their workspace, and talk to each other.

In the cooperative task, participants completed the Jigsaw Phrase Guessing game together as a team. They could share their partner’s progress and help the partner move pieces or guess the solution. The round was complete when both participants had found the correct two-word phrase. If they completed the round within 2 minutes, each participant was rewarded with 100 credits. (Note: this 2 minutes threshold was only to motivate participants to complete task as soon as possible. They still needed to complete each round even if they exceeded 2 minutes)

In the competitive task, the goal was to be the first to correctly guess the two-word phrase. Participants might monitor their partner’s progress and take advantage of peeking to get a hint of how to assemble the pieces or in order to guess the phrase first. The winner was decided when one of the participants shouted out the correct two-word phrase. If they completed the round within 2 minutes, the fastest individual was rewarded with 200 credits.

**Incentive and risk** In order to incentivize participants to earn as great a number of credits as possible, the experimenter told the participants that they would receive an extra £0.10 for every 100 credits that they received. At the end of the study, regardless of their performance on the task, all participants received £5.00, which was more than they could earn in the game. These uniform payments were requested by a reviewer for University College London Research Ethics Committee.

#### Design

The study had a 3 embodiment types (no self-avatar, self-avatar, or face to face) × 2 collaboration styles (competitive or collaborative), resulting in 6 between-subject conditions. Subjects were randomly assigned to the between-subject conditions. To increase the sample size, we additionally asked each subject pairs to complete 5 rounds of Daytrader game, and 10 rounds of Jigsaw Phrase Guessing game as within-subjects conditions.

#### Procedure

**Fig 5:**
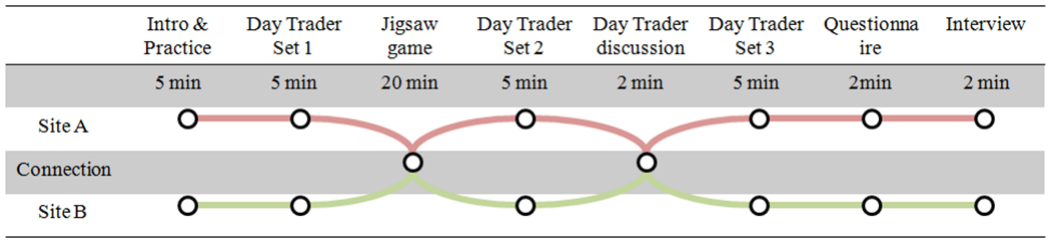
The timeline of the experimental protocol.

The timeline of the protocol is illustrated in Fig 5.

Before beginning the experiment, participants at both sites were asked to fill out a brief demographic survey as well as a consent form. The experimenters instructed the participants on the structure of the Daytrader game and provided the participants with an overview of the Jigsaw Phrase Guessing game that the participants would engage in. They then guided the participant on how to manipulate objects using controllers in the VR conditions, or explained the rules of manipulating objects using hands in the real word conditions.

Following practice, the experimenters at both sites instructed the participants to complete the first set of Daytrader Game rounds. They could not to speak each other while playing. Next, the experimenters at both sites set up an audio call via Skype and put participants into the virtual reality by helping them don the HMD; or arranged participants to move into the same room. After the connection were established, participants were asked to complete 10 rounds of jigsaw puzzles in one of six experimental conditions. Next, the experimenters at both sites terminated the connection, and the participants will engage in the second set of Daytrader Game rounds. After this, the participants were connected and given 2 minutes to discuss their strategy for the Daytrader game. Then, the participants were disconnected for the third set of Daytrader Game rounds. Then, the experimenters at both sites administered a post-experiment questionnaire, and conducted a debrief interview.

Upon completion of the experiment, the participants were paid £5.00 for their participation. The experiment took about 45 minutes.

#### Scoring

We measured the collaboration outcomes by using a number of objective, behavioural, and subjective measures, as described in the paragraphs below.

**Jigsaw game completion time** Task completion time was defined as the slower of the two participants’ time to find the correct two-word phrase in the cooperative condition, and as the faster of the two participants’ time to find the correct two-word phrase in the competitive condition.

**Trust** Similar to previous work, we examined the cooperative investments, the credits that dyads put in the shared pool, as a measure of trust. For example, for the first round, the measure of trust can be from 0 (both participants invest nothing) to 60 (both participants invest all their credits).

In the first set of rounds, the participants were told only that the person with whom they were playing was also a study participant. Thus, the cooperative investments in the first set measured the trust that participants had for a stranger. The participants played the second set of the Daytrader Game after they completed the Jigsaw Phrase Guessing game. Thus, the difference between the cooperative investments in the first and second sets indicated the amount of trust gained from their interaction with the other person. The participants played the third set after they had discussed and formed a strategy for the last set together. Thus, the cooperative investments for the last set measured the fragility of the trust developed between participants.

**Post-questionnaire** We used a truncated version of the Specific Interpersonal Trust Scale [34] to measure subjective trust between participants. This version contained 4 statements about the other person such as “I would expect the other person to pay me back if I loaned him/her £100.”, and “If the other person gave me a compliment on my haircut I would believe s/he meant what was said.” The participants responded on 7-point Likert scales with the anchor 1 (Strongly disagree)—7 (Strongly agree).

**Post interview & observations** We interviewed each participant separately. There were no predetermined questions, but the topics covered were general impressions of the other participant, any specific incidents in the game that stood out (e.g. why s/he felt lied to, etc), and discussion of any strategies they used for each stage of the experiment (e.g. how s/he decide the cooperative investments before and after playing the jigsaw game). The post interview was to help explain some observed events during the game and to guide future research.

### Result

Table 1 Gave a summary of measurement results, including the Jigsaw game completion time, and the final cooperative investments for each set of Daytrader Game.

**Table 1:** Summary of measurement results.

Embodiment	No Self-Avatar		Self-Avatar		Face to Face	
collaboration styles	Cooperative	Competitive	Cooperative	Competitive	Cooperative	Competitive
*Jigsaw game completion time (M)*	55.01	47.99	41.6	64.87	31.83	49.59
*Jigsaw game completion time (95% CI)*	(44.2, 65.82)	(37.18, 58.79)	(30.79, 52.41)	(54.07, 75.68)	(21.02, 42.63)	(38.78, 60.395)
*Set 1 final cooperative investments (M)*	864.5	814.75	550	817.25	733.75	1095.25
*Set 1 final cooperative investments (SD)*	346.73	994.38	570.09	797.42	908.22	777.104
*Set 2 final cooperative investments (M)*	612.5	516	2879.5	1736	1878.5	2155.25
*Set 2 final cooperative investments (SD)*	342.48	515.5	1609.65	1391	1956.29	1425.13
*Set 3 final cooperative investments (M)*	858	784.25	3571.75	2867.5	4408.75	2918.75
*Set 3 final cooperative investments (SD)*	379.55	969.33	1561.21	2300.83	526.5	1504.99

#### Jigsaw game performance

**Fig 6:**
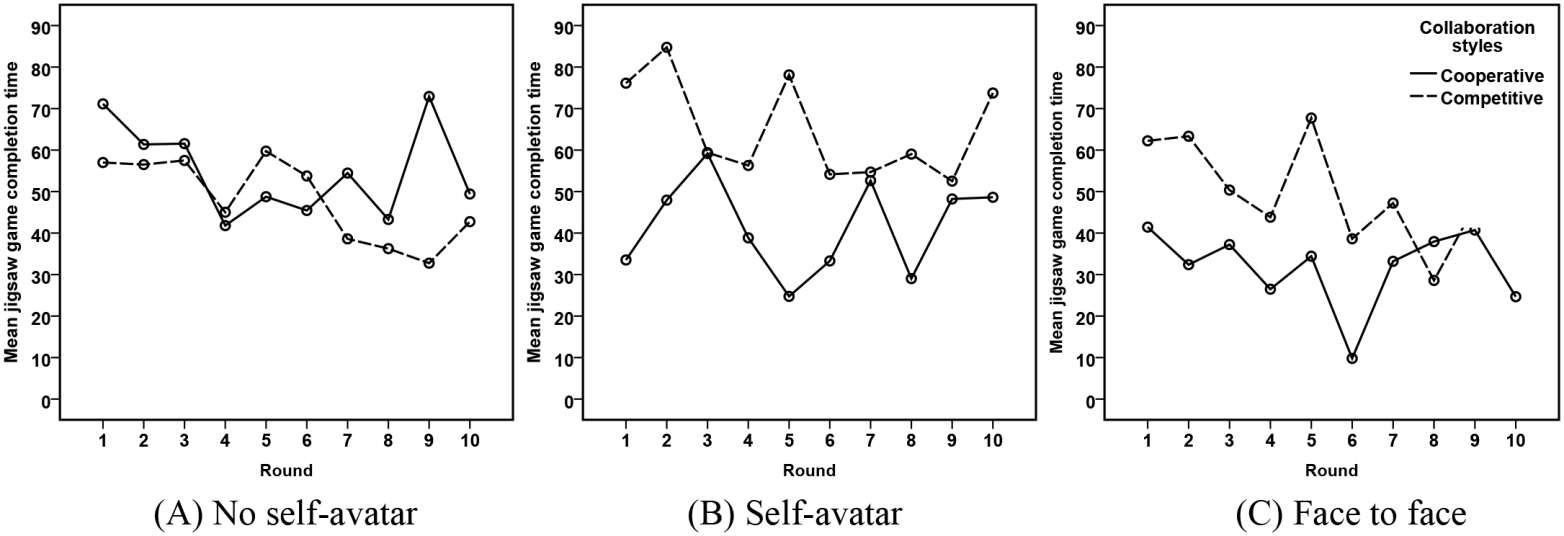
Jigsaw game completion time for three embodiment levels.

Fig 6 presents the mean Jigsaw Game completion time for different embodiment levels. We can see that participants took more time completed the task in a competitive style (the dashed line) than they did using a cooperative style (the solid line), in the self-avatar condition and the face to face condition. However, no such difference was presented in the face to face condition.

A mixed ANOVA was conducted on the mean completion time, with embodiment levels and collaboration styles as between-subjects factors and 10 rounds as a within-subjects factor. There were no outliers in the data, as assessed by inspection of a boxplot. The completion time were normally distributed, as assessed by a Shapiro-Wilk test (*p* > .05). There was homogeneity of variances, as assessed by Levene’s test for equality of variances (*p* > .05). Mauchly’s test of sphericity indicated that the assumption of sphericity had been violated, (*χ*^2^(44) = 62.344, *p* = .046), therefore degrees of freedom were corrected using Greenhouse-Geisser estimates of sphericity (*ϵ* = .531). The main effect of rounds was not significant, *F*(4.779, 86.03) = 1.693, *p* = .148. The main effect of embodiment levels was not significant, *F*(2, 18) = 3.484, *p* = .053. However, the main effect of collaboration styles was significant, *F*(2, 18) = 7.289, *p* = .015. The embodiment levels × collaboration style interaction was significant, *F*(2, 18) = 4.923, *p* = .02.

We then looked at the completion time for each collaboration style. Results showed a significant main effect of embodiment levels on performance in cooperative collaboration (*F*(2, 9) = 10.213, *p* = .005), but not in competitive collaboration (*F*(2, 9) = 2.193, *p* = .168). H3a and H4a are not supported. Bonferroni corrected comparisons of cooperative condition showed participants completed the game significantly slower in the no self-avatar condition than the self-avatar condition (*p* = .029) and face to face condition(*p* = .001). H1a is supported. It also showed there were no significant differences between the no-avatar condition and the face to face condition (*p* = .09). H2a is not supported.

We further asked whether the completion time were affected differently by embodiment levels. Results revealed that participants completed the game significantly faster in a cooperative style than they did using a competitive style for both the self-avatar condition, *F*(1, 6) = 9.107, *p* = .023, and the face to face conditions, *F*(1, 6) = 6.776, *p* = .04, but not for the no self-avatar condition, *F*(1, 6) = 0.937, *p* = .371. Thus, H6a and H7a are supported, but H5a is not supported.

#### Trust

**Fig 7:**
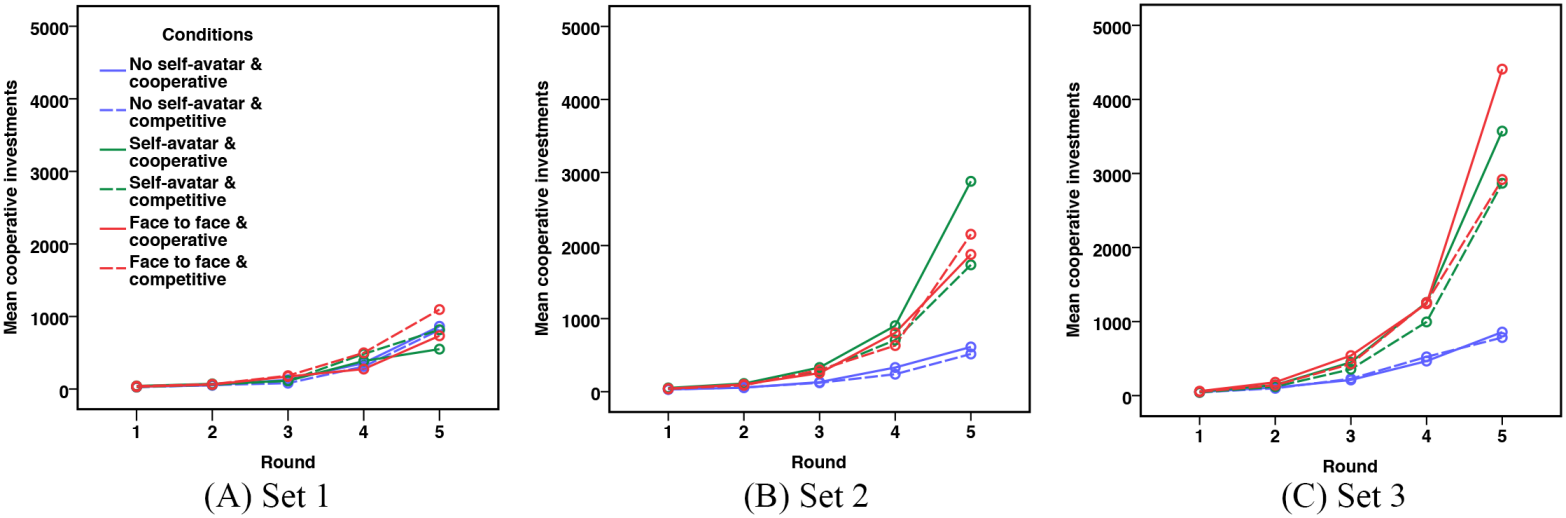
Mean cooperative investments made round-by-round.

We took a look at the cooperative investments made round-by-round. Fig 7 showed the mean cooperative investments for in each of the six between subject conditions at three sets of Daytrader Game. Table 1 shows the mean cooperative investments for the last round at three sets of Daytrader Game. Descriptive results showed participants in the self-avatar condition and the face to face condition showed a significant increase in trust after interacting, compared with the no-avatar condition.

A mixed ANOVA was conducted on the mean cooperative investments, with embodiment levels and collaboration styles as between-subjects factors, and set and round as within-subjects factors. There were no outliers in the data, as assessed by inspection of a boxplot. The cooperative investments were normally distributed, as assessed by a Shapiro-Wilk test (*p* > .05). There was homogeneity of variances, as assessed by Levene’s test for equality of variances (*p* > .05). Firstly, we looked at the main effect of three sets of Daytrader Game. Mauchly’s test of sphericity indicated that the assumption of sphericity was met, (*χ*^2^(2) = 1.792, *p* = .408). There was a statistically significant difference in the mean cooperative investments, *F*(2, 36) = 43.898, *p* < .001. Bonferroni post-hoc tests revealed significant differences among each set of Daytrader Game. The set × embodiment levels interaction was significant, *F*(4, 36) = 9.228, *p* < .001. However, set × collaboration styles interaction and set × embodiment levels × collaboration styles were not significant, with *F*(2, 36) = 2.226, *p* = .123 and *F*(4, 36) = 1.235, *p* = .313, respectively. These results indicated that the mean cooperative investments due to embodiment levels was different in three sets of Daytrader game. Secondly, the main effect of collaboration styles was not significant, *F*(1, 18) = .343, *p* = .566. The collaboration styles × embodiment levels interaction was also not significant, *F*(2, 18) = .076, *p* < .927. Thus, H5b, H6b, and H7b are not supportted.

For the first set of the Daytrader Game (see Fig 7A), results revealed that the main effect of embodiment levels, the main effect of collaboration styles, the embodiment levels × collaboration styles interaction were all not significant, with *F*(2, 18) = .097, *p* = .908, *F*(1, 18) = .283, *p* = .601, *F*(2, 18) = .192, *p* = .827, respectively. As expected, before jigsaw game interaction, there were no significant differences among the three embodiment levels, with respect to trust between participants.

For the second set of the Daytrader Game (see Fig 7B), results showed that the mean cooperative investments differed significantly across three embodiment levels, *F*(2, 18) = 3.894, *p* = .039. However, the main effect of collaboration styles, and the embodiment levels × collaboration styles interaction were not significant, with *F*(1, 18) = .393, *p* = .538, and *F*(2, 18) = .356, *p* = .705, respectively. All pairwise comparisons were run with reported 95% confidence intervals and p-values are Bonferroni-adjusted. The mean cooperative investments in the no self-avatar condition was lower than the self-avatar and the face to face condition, with statistically significant mean differences of −501.525 (95% *CI*, [−905.564, −97.486]), *p* = .018 and −416.325 (95% *CI*, [−820.364, −12.286]), *p* = .044. However, the self-avatar condition did not significantly differ from the face to face condition, with a mean difference of 85.2 (95% *CI*, [−318.839, 489.239]), *p* = .663. These results indicate that the jigsaw game interaction did affect how much participants were able to make in the Daytrader game. Thus, H1b and H3b were supported, but H2b and H4b were not supported.

For the third set of the Daytrader game (see Fig 7C), the mean cooperative investments also differed significantly across three embodiment levels, *F*(2, 18) = 8.208, *p* = .003. However, the main effect of collaboration styles, and the embodiment × collaboration styles interaction were not significant, with *F*(1, 18) = 1.169, *p* = .294, and *F*(2, 18) = .316, *p* = .733, respectively. All pairwise comparisons were run where reported 95% confidence intervals and p-values are Bonferroni-adjusted. The mean cooperative investments in the no self-avatar condition was lower than the self-avatar and the face to face condition, with statistically significant mean differences of −648.725 (95% *CI*, [−1083.815, −213.635]), *p* = .006 and −785.225 (95% *CI*, [−1220.315, −350.135]), *p* = .001; however, the self-avatar condition did not significantly differ from the face to face condition, with a mean difference of −136.5 (95% *CI*, [−571.59, 298.59]), *p* = .518.

#### Post-questionnaire

For each session, the responses to each questionnaire item given by the participant from both sites were averaged to create an aggregate session response. We calculated Cronbach’s *α* as the reliability test for the questionnaire (4 items, *α* = .856).

**Fig 8:**
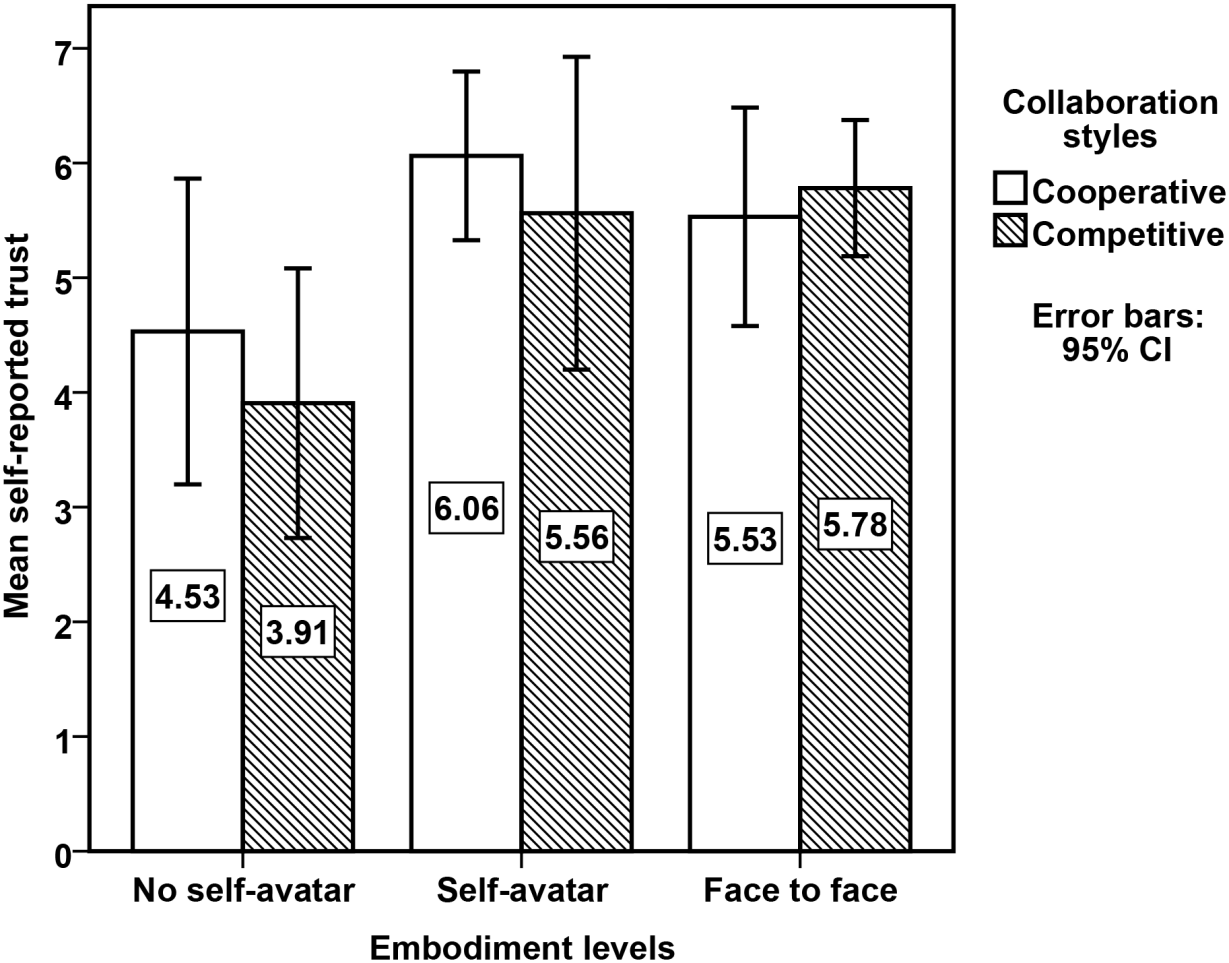
Self-reported trust for different embodiment levels and collaboration styles.

We then compared self-reported trust measures by embodiment levels and collaboration styles. The means are presented in Fig 8. A 3 × 2 between-subjects ANOVA was conducted on the participants’ self-reported score. There were no outliers, as assessed by inspection of a boxplot. Data was normally distributed, as assessed by Shapiro-Wilk’s test (*p* > .05). There was homogeneity of variances, as assessed by Levene’s test for equality of variances, *p* = .395. The main effect of embodiment levels was significant, *F*(2, 18) = 13.758, *p* < .001. Tukey HSD post-hoc tests revealed that no avatar condition self-reported significantly lower trust than the self-avatar condition, *p* < .001 and the face to face condition, *p* = .001. No significant difference in self-reported trust was found between the self avatar condition and the face to face condition, *p* = .888. Secondly, the main effect of collaboration styles not was significant, *F*(1, 18) = 1.137, *p* = 0.3. Thirdly, the embodiment levels × the collaboration styles interaction not was significant, *F*(2, 18) = .998, *p* = .388. Thus, the results of these comparisons match the results of the comparisons of trust from the Daytrader measurements.

#### Post interview & observations

**Fig 9:**
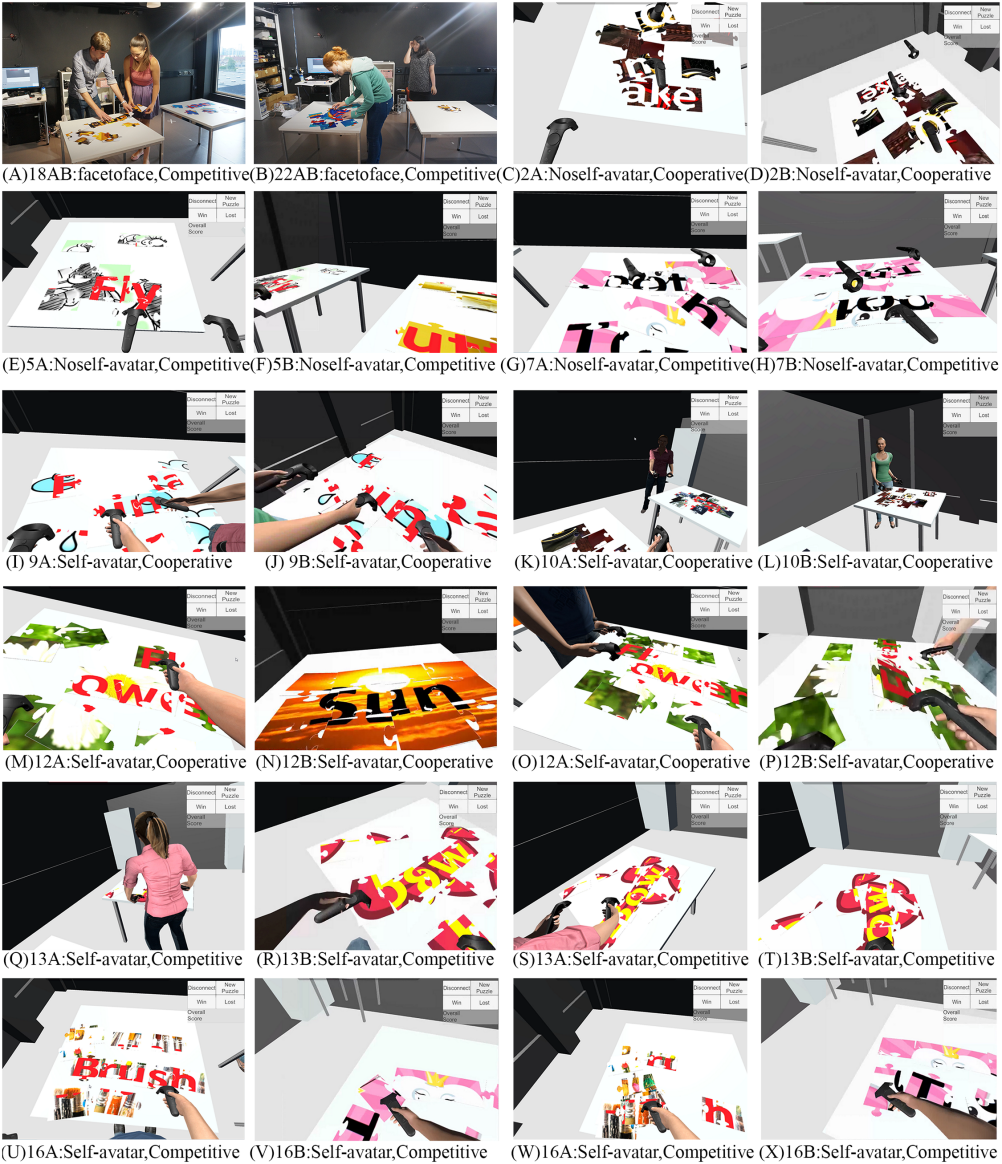
Various communicative acts for different conditions. Each pair of screenshots was simultaneously captured from the first-person view of each participant within the dyad. Note: 18AB = dyad number 18, participant at site A, and participant at site B.

We also observed differences in how participants interacted during each condition. In particular, we analyzed transcripts from our interviews and video footage, focusing on how participants collaborated. As described below, Fig 9 shows various communicative acts for different conditions. Participants are labeled with dyad number and sites in the succeeding text. For example, participant 2A indicated dyad number 2 at site A.

**Workspace** As soon as the participants started, we saw that they predominantly separated and went to two tables, each claiming their respective workspace. None of them appeared to plan to work in a tightly coupled fashion, where they could choose to stand next to each other in front of the same table at the beginning. This occurred naturally across all participants, without requiring any verbal negotiation.

For the collaborative tasks, we observed several cases in which a participant who had completed their part of the puzzle moved to the partner’s work space and assisted their partner (12B, see Fig 9M–9P). For the self-avatar condition, we saw the avatar might more easily convey a sense of on-going activity (8A and 9B, see Fig 9I and 9J). For example, the limbs can more accurately indicate which puzzle piece a given user is currently accessing or is going to access. On the other hand, due to the lack of visibility in the no self-avatar condition, We observed quite a few cases where users tried to access the same puzzle piece. One participant directly commented: “He always try to take control of my pieces. Not really helping.” Thus, this introduces a possibility of interference and confusion, where one person’s actions potentially disturb the productivity of others.

For the competitive tasks, from the observations of all trials in the face to face condition and the self-avatar condition, participants never picked the puzzle pieces in their partner’s workspace, and they normally only glanced at their partner’s workspace to check their progress. One participant stated: “I don’t want to be that aggressive.” Perhaps the social and behavioral norms defining personal territories may still be in effect. By contrast, in the no avatar condition, we occasionally saw a participant intruding to the partner’s work space and manipulated their pieces (7A and 7B, see Fig 9G and 9H).

**Body orientation** While participants were cooperating at the same table, we observed several notable differences in participant body orientations. For the face to face condition and the self-avatar condition, participant often stood side-by-side, and tilted their bodies toward each other (18A and 18B, see Fig 9A; 9A and 9B, see Fig 9I and 9J). However, for the no self-avatar condition, participants seemed more evenly distributed (2A and 2B, see Fig 9C and 9D).

**Hiding information behavior** During the competitive tasks, participants still wanted to view their partner’s information but were not willing to share information with their partner. One of the most interesting observations was that participants often used their body to occlude their personal information. We found that this occurred in 23 out of 40 cases in the face to face condition (22AB, see Fig 9B) and 21 out of 40 cases in the self-avatar condition (13A, see Fig 9Q). In these cases, their partner needed to walk around to view the information (see Fig 9S). This contrasts with no self-avatar condition where, in general, simply by turning their head, all information is viewable to all participants from all locations at all times (see Fig 9E and 9F).

Another interesting observation was that after completing their part of the puzzle (16A, see Fig 9U), participants would quickly mess up the result to avoid their partner view the information (see Fig 9W). This consistently happened across all conditions during the competitive tasks. Also, comparing to the cooperative situation, participants tried to minimize the amount of their own workspace obscure their information (see Fig 9B).

**Nonverbal communication** In the face to face and the self-avatar condition, we observed the participants making several other communicative actions such as looking at each other (see Fig 9K and 9L), nodding, shrugging and waving good-bye.

**Verbal communication** During the collaborative tasks, some groups maintained a conversation while collaborating, constantly updating each other on the visible portions of their word, guesses of what their word or phrase might be, and strategies for finding their words. Some groups didn’t feel a need to constantly update the partner verbally on progress, as a quick glance was sufficient for sharing the partner’s work. One participant (9B) in the self-avatar condition commented: “we can see each other, we don’t necessarily have to communicate verbally all the time.”

To ensure the other partner could clearly understand their advice, participants in the no avatar condition gave detailed instructions and described specific pieces while pointing. In contrast to deictic references such as “this” or “that”, which were more frequently observed in the self-avatar condition and the face to face condition.

However, no groups used verbal communication during competitive tasks.

**Strategies for the daytrader game** Perhaps the biggest surprise is when asked directly “Did you feel like investing more credits after playing the jigsaw game?”, 44 participants reported yes to some extent, two could not say, and two reported no (one in no self-avatar collaborative condition, and one in face to face competitive condition). In the words of one participant (14A, with a self-avatar, competitive): “After the jigsaw game, I feel she closer to me 〈*sic*〉. So I tend to trust her more.”

## Discussion

On the basis of the above findings, we will now revisit the hypotheses set out earlier. The results on the game completion time support H1a, H6a, and H7a. The results of the trust formation analysis support H1b and H3b. The rest of them are not supported, but this is not surprising in retrospect.

As we expected, participants could finish the task faster when they cooperated than when they competed in both face to face condition and the self-avatar condition. However, the collaboration styles did not significantly affect task performance in the no self-avatar condition. We explain this by our observations that participant often used their body to hide their information during the competition in both face to face condition and the self-avatar condition. On the other hand, in the no self-avatar condition, participants could easily see the partner’s workspace by turning their head. Also, this result may be explained by the fact that participants were more aggressive in the no self-avatar condition.

When analyzing the effects of the utility of a self-avatar in detail, by separating cooperative and competitive tasks, we found that the self-avatar condition was superior to the no self-avatar in the cooperative task. This result reinforces the validity of previous SVE studies (e.g., [18]) that indicate that including self-avatars should help users perform tasks more accurately and/or quickly in an HMD VE. As for the competitive task condition, contrary to expectations, embodiment levels had no significant effect on performance. This suggested that easy peeking of information in the no self-avatar might compensate for decrease in accuracy and increase in completion time.

Regarding trust formation, our results showed that participants completing the jigsaw game with a self-avatar gained more trust than without a self-avatar. This result might be explained by several factors. For example, the self-avatar could embody the user, and increase social presence and interpersonal trust in avatar mediated communication [32]. Another possible reason might be due to participants often tilted their bodies toward each other in the self-avatar condition during cooperation, indicating that they belonged to the same group and shared a higher sense of unity [33].

Interestingly, the collaboration style did not significantly affect trust between participants. Due to the choice of tasks, some researchers found that a cooperative task may invoke positive attitudes and behaviour toward collaborators, whereas a competitive task may evince negative attitudes and behaviours toward competitors. For example, Ewoldsen et al. found that people who played a violent video game cooperatively were more likely to work together on a subsequent task than those who played a violent video game competitively [31]. However, others suggested that competition and cooperation did not differ. For example, Vang et al. investigated the influence of an avatars’ race and task collaboration to determine how users would perceive others in a virtual world [30]. They found after performing either cooperative task or competitive task could lead white users to evaluate black avatars positively. In our case, sharing a task, regardless of its goal, led to more interpersonal trust between participants.

There are several potential avenues for next steps. Firstly, advances in consumer head-mounted displays will make it possible for us continually evolve our SVE system to best leverage new capabilities. For example, the FOVE virtual reality HMD provides eye tracking capabilities. Li et al. developed a HMD that enabled 3D facial performance-driven animation in real-time [37]. Because nonverbal cues, such as eye gaze, facial expression and deictic gestures, provide important additional channels of information during communication [35, 36, 38, 39], we plan to using these novel HMDs to improve our SVE system. Secondly, we hope to leverage our system for 3-way or N-way teleconferencing scenarios. Thirdly, the choice of an avatar and its animation techniques might influence collaborative outcomes. For example, different appearances of avatars might result in different impression formation and activation of social stereotypes. Also, we plan to add another condition, using different avatar body (e.g., skinny avatar), such that participants are not ‘peeking’ or occluding each other’s view, to further demonstrate our results.

## Conclusion

We implemented an SVE system in which the participants in separate physical space could have the opportunity to embody an avatar and interact in an SVE. We provided each user with a HTC Vive HMD and controllers in order to view and manipulate the virtual world. Our system featured a client-server architecture that is scalable and easy to use.

In an experiment we compared performance in a two-person game among three conditions: without self-avatar, with self-avatar, as well as face to face conditions. Additionally, we included two collaboration styles: competitive and cooperative tasks.

Results demonstrated that users in the self-avatar condition completed the task more quickly that users in no self-avatar in cooperative tasks; however, embodiment levels had no significant effect in competitive tasks. Additionally, participants completed the task faster in a cooperative style than they did using a competitive style for the self-avatar and the face to face condition. However, interestingly we were not able to find such effect in the no self-avatar condition. Furthermore, participants with a self-avatar showed a significant increase in trust after interaction, compared with participants without a self-avatar. Surprisingly, after sharing a task, regardless of its goal, the amount of trust built between participants could be increased. We observed various communicative acts, such as, participants using their body to occlude the personal information during competitive task, tilting their bodies toward each other during collaborative task etc. These similar behaviours still hold in immersive virtual reality, but the self-avatar is necessary. Lastly, users’ self-reports also supported these findings.

To conclude, our results have important implications for the design of virtual reality HMD systems: simply adding a self-avatar, without changing any hardware configuration, could increase communicative and collaborative outcomes in a SVE.

## Supporting information

S1 File Original questions. (PDF)

S2 File Information Sheet and Consent Form. (PDF)

S3 File Data. (XLSX)
