# Supplementary material for: The impact of self-avatars on trust and collaboration in shared virtual environments
Source: PLoS One. 2017 Dec 14;12(12):e0189078. doi: 10.1371/journal.pone.0189078 (PMC5730128; doi:10.1371/journal.pone.0189078)
Supplement: S1 File — (PDF) [file pone.0189078.s001.pdf]

Number:

What gender do you identify as?
How old are you?
What is your occupation/field of study?
How familiar are you with virtual reality system?

I would expect the other person to pay me back if I loaned him/her $100.

1 2 3 4 5 6 7

If the other person laughed unexpectedly at something I did or said, I would know s/he was not being unkind.
1 2 3 4 5 6 7

If the other person gave me a compliment on my haircut I would believe s/he meant what was said.
1 2 3 4 5 6 7

If the other person borrowed something of value and returned it broken, s/he would offer to pay for the repairs.
1 2 3 4 5 6 7

Additional comments:
