# Supplementary material for: The impact of self-avatars on trust and collaboration in shared virtual environments
Source: PLoS One. 2017 Dec 14;12(12):e0189078. doi: 10.1371/journal.pone.0189078 (PMC5730128; doi:10.1371/journal.pone.0189078)
Supplement: S2 File — (PDF) [file pone.0189078.s002.pdf]

Information Sheet for Participants

You will be given a copy of this information sheet.

Title of Project: **Trust and embodiment for immersive virtual reality system**

This study has been approved by the UCL Research Ethics Committee (Project ID Number):

Name	**Anthony Steed,**
Work Address	**Department of Computer Science, University College London, Gower Street, London WC1E 6BT**
Contact Details	**A.Steed@ucl.ac.uk**

We would like to invite you to participate in this research project.

Details of Study:

The purpose of the study is to investigate the trust and embodiment of current virtual reality systems. You will complete a series of games that are played with another participant. You will use head-mounted display systems.

The whole experiment should take approximately 30 minutes.

If you have any questions about the study now please ask the experimenter. If you have any questions at a later date, please email Anthony Steed at the addresses above.

IMPORTANT

When people use virtual reality systems, some people sometimes experience some degree of nausea. If at any time you wish to stop taking part in the study due to this or any other reason, please just say so and we will stop.

There has been some research, which suggests that people using head-mounted displays might experience some disturbances in vision afterwards. No long term studies are known to us, but the studies which have been carried out do testing after about 30 minutes, and find the effect is still sometimes there.

There have been various reported side effects of using virtual reality equipment, such as 'flashbacks'.

With any type of video equipment there is a possibility that an epileptic episode may be generated. This, for example, has been reported for computer video games or television viewing.

Please Turn Over

Procedure:

- You will be asked to read, understand and sign a **Consent Form**. If you agree to take part in this experiment then we will ask you sign it, and the study will continue with your participation. Otherwise your involvement will cease at that point. **Note that in any case you can withdraw at any later time without giving any reasons.**
- You will be asked to switch off mobile phones during the experiment.
- You will be asked to complete a demographics questionnaire.
- The main experiment will involve using a head-mounted display and going though the following stages:
    - Practice using the head-mounted display
    - Play one round of Day Trader game with another player (the Day Trader game is explained below)
    - Complete a Jigsaw Puzzle game (the Jigsaw Puzzle game is explained below)
    - Play a second round of the Day Trader game with another player
    - Discuss the Day Trader game with the other player
    - Play a third round of the Day Trader game
- You will then take off the head-mounted display and complete the following tasks:
    - Post-Experiment Questionnaire
    - Brief interview

  [Day Trader game] During a round, each participant will be given 30 credits that they can either keep or put into a pool that was shared between the two participants. At the end of the round, credits that they chose to keep will doubled in value, while the tokens in the shared pool will be tripled and then split evenly between the two participants.

  At the end of each set of five rounds, the participant that earns the most credits in that set will receive a 300 credits bonus. If both participants earned the same amount, they both received the bonus.

  We will explain the rules of the Day Trader game again before you put on the head-mounted display.

  [Jigsaw Puzzle game] The goal of our jigsaw puzzle game is to find a hidden two-word phrase inside two solution images. Each solution image contains a different word, which together form a phrase. There are two components to completing a game: finding a word and finding a phrase. When each player moves a piece to the correct position, this piece might reveal a portion of a word.

  We will explain the rules of the Jigsaw Puzzle game again before you put on the head-mounted display.

- You will be paid £5 in cash for your participation.
- Because this study is ongoing, please do not discuss it with others for about **three months**.
- **Thank you** for your participation.

Please Turn Over

Note:

- A decision to withdraw at any time, or decision not to take part, will not affect the standard of care you receive.
- You may withdraw your data from the project at any time up until it is transcribed for use in the final report on the 15th September 2016.
- We will record your name and assign you a participant number. A record matching your name and participant number will be made on a piece of paper separate from all other data collection means and kept in a locked cabinet. The reason for keeping this record is so that we can facilitate removal of your data from the project as stated above. This record will be destroyed by shredding the relevant paper on or shortly after 16th September 2016, so that only anonymous data records are retained. This anonymous data is that data that we are asking your permission to retain for writing reports and for future research projects.
- If you decide to take part you will be given this information sheet to keep and be asked to sign a consent form.

Please discuss the information above with others if you wish or ask us if there is anything that is not clear or if you would like more information.

It is up to you to decide whether to take part or not; choosing not to take part will not disadvantage you in any way. If you do decide to take part you are still free to withdraw at any time and without giving a reason.

All data will be collected and stored in accordance with the Data Protection Act 1998.

Informed Consent Form

Please complete this form after you have read the Information Sheet and/or listened to an explanation about the research.

Title of Project: **Trust and embodiment for immersive virtual reality system**

This study has been approved by the UCL Research Ethics Committee (Project ID Number):

Thank you for your interest in taking part in this research. Before you agree to take part, the person organising the research must explain the project to you.

If you have any questions arising from the Information Sheet or explanation already given to you, please ask the researcher before you to decide whether to join in. You will be given a copy of this Consent Form to keep and refer to at any time.

Participant's Statement

I

- have read the notes written above and the Information Sheet, and understand what the study involves.
- understand that if I decide at any time that I no longer wish to take part in this project, I can notify the researchers involved and withdraw immediately.
- consent to the processing of my personal information for the purposes of this research study.
- understand that such information will be treated as strictly confidential and handled in accordance with the provisions of the Data Protection Act 1998.
- agree that the research project named above has been explained to me to my satisfaction and I agree to take part in this study.
- I understand that the information I have submitted will be published as a report and I may request a copy. Confidentiality and anonymity will be maintained and it will not be possible to identify me from any publications.
- I agree that my non-personal research data may be used by others for future research. I am assured that the confidentiality of my personal data will be upheld through the removal of identifiers.
- I certify that I do not have epilepsy.
- I certify that I have not consumed alcohol within the last 6 hours.

Signed: Date:
